# Cerebral blood flow and vasoreactivity in aging: an arterial spin labeling study

**DOI:** 10.1590/1414-431X20175670

**Published:** 2017-03-23

**Authors:** R.F. Leoni, I.A.F. Oliveira, O.M. Pontes-Neto, A.C. Santos, J.P. Leite

**Affiliations:** 1 Departamento de Física, Faculdade de Filosofia, Ciências e Letras de Ribeirão Preto, Universidade de São Paulo, Ribeirão Preto, SP, Brasil; 2 Departamento de Neurociências e Ciências do Comportamento, Faculdade de Medicina de Ribeirão Preto, Universidade de São Paulo, Ribeirão Preto, SP, Brasil; 3 Divisão de Radiologia, Departamento de Clínica Médica, Faculdade de Medicina de Ribeirão Preto, Universidade de São Paulo, Ribeirão Preto, SP, Brasil

**Keywords:** Arterial spin labeling, Blood oxygenation level-dependent contrast, Cerebral blood flow, Cerebrovascular reactivity, Hypercapnia, Magnetic resonance imaging

## Abstract

Regional cerebral blood flow (CBF) and cerebrovascular reactivity (CVR) in young and elderly participants were assessed using pulsed arterial spin labeling (ASL) and blood oxygenation level-dependent (BOLD) magnetic resonance imaging (MRI) techniques in combination with inhalation of CO_2_. Pulsed ASL and BOLD-MRI were acquired in seventeen asymptomatic volunteers (10 young adults, age: 30±7 years; 7 elderly adults, age: 64±8 years) with no history of diabetes, hypertension, and neurological diseases. Data from one elderly participant was excluded due to the incorrigible head motion. Average baseline CBF in gray matter was significantly reduced in elderly (46±9 mL·100 g^-1^·min^-1^) compared to young adults (57±8 mL·100 g^-1^·min^-1^; P=0.02). Decreased pulsed ASL-CVR and BOLD-CVR in gray matter were also observed in elderly (2.12±1.30 and 0.13±0.06 %/mmHg, respectively) compared to young adults (3.28±1.43 and 0.28±0.11 %/mmHg, respectively; P<0.05), suggesting some degree of vascular impairment with aging. Moreover, age-related decrease in baseline CBF was observed in different brain regions (inferior, middle and superior frontal gyri; precentral and postcentral gyri; superior temporal gyrus; cingulate gyri; insula, putamen, caudate, and supramarginal gyrus). In conclusion, CBF and CVR were successfully investigated using a protocol that causes minimal or no discomfort for the participants. Age-related decreases in baseline CBF and CVR were observed in the cerebral cortex, which may be related to the vulnerability for neurological disorders in aging.

## Introduction

Aging has been associated with motor changes and cognitive decline that can seriously interfere with quality of life (1,2). In addition, age-related cerebrovascular alterations have been reported (3,4). Therefore, the study of cerebral perfusion in advanced age is important to understand neurovascular mechanisms that underlie the reported changes and their relationship with age-related pathologies. Thus, noninvasive assessment of cerebral blood flow (CBF) and cerebrovascular reactivity (CVR) may provide additional information about the integrity of cerebrovascular reserve.

Magnetic resonance imaging (MRI) techniques, such as arterial spin labeling (ASL) and blood oxygenation level-dependent (BOLD) contrast, have been widely used in combination with vasoactive challenges to map CVR, such as acetazolamide (ACZ) administration, breath-holding test (BHT) and CO_2_ inhalation (5). Because ACZ administration is invasive and BHT is highly dependent on participant's collaboration, CO_2_ inhalation is an attractive alternative. CO_2_ causes great but reversible dilation of cerebral arteries and arterioles, and increased CBF. Although different mechanisms have been proposed to explain the relationship between hypercapnia and CBF, the decrease in perivascular pH appears to be the main mechanism (6). Because the resistance of smooth muscles of blood vessels is sensitive to regional pH variation, hypercapnia results in a decrease of cerebrovascular resistance, and consequently global vasodilation. However, other mechanisms involving nitric oxide and prostanoids were reported to contribute to hypercapnic cerebral vasodilation, but they seem to be specific to different species (7). Therefore, an increase in arterial partial pressure of CO_2_ (PaCO_2_) leads to an increase in global CBF, due to increased vascular blood velocity (8). Moreover, the CBF response to hypercapnia may vary across brain regions, but hypercapnia does not significantly alter cerebral metabolism (9).

Although BOLD-MRI has been extensively used in functional studies and also for CVR assessment in combination with a hypercapnic challenge (10–13), its signal depends on a complex relationship between CBF, cerebral blood volume, and oxygen extraction. In contrast, ASL technique uses blood water as an endogenous contrast resulting in an entirely noninvasive alternative to assess cerebral blood flow quantitatively (14–17), which makes it particularly useful for cerebrovascular reserve assessment in longitudinal studies (14,18,19). Among different ASL methods, pulsed ASL uses a single, short, adiabatic radiofrequency pulse to label a large volume adjacent to the image plane, resulting in a high labeling efficiency and low energy deposition, but also, a low signal-to-noise ratio (SNR) (15 [Bibr B16]). Commonly, pulsed ASL measures are conducted with a single post-labeling delay (PLD) between the labeling and the image acquisition. Then, they rely heavily on the arterial arrival time (AAT), resulting in errors on CBF quantification if PLD is not chosen appropriately (20). Considering the well-known AAT changes with aging, an adequate PLD for each subject group was chosen in the present study as recommended by the ISMRM Perfusion Study Group and the European Consortium for ASL in Dementia (21).

Although studies have reported lower baseline CBF and CVR levels in elderly compared to young adults, recent studies using ASL have reported different results in aging: global gray matter CBF reduction (22,23), no global gray matter CBF reduction (4,24), or region-specific gray matter CBF alterations (2,3,25). In addition, few CVR assessments have been done using ASL and CO_2_ inhalation in elderly people (1,26). Despite their disadvantages, BOLD-MRI or transcranial Doppler ultrasound in combination with breath-holding is more common due to their clinical availability and easiness to implement (12,13).

Therefore, in the present study, pulsed ASL and BOLD-MRI techniques were combined with inhalation of CO_2_ to assess age-related effects on regional CBF and CVR in healthy participants. We investigated changes in CBF and BOLD response to CO_2_ using an experimental setup that minimizes participant's discomfort, and MRI sequences available for clinical routine.

## Material and Methods

### Participants

Seventeen asymptomatic volunteers (10 young adults, age: 30±7 years; 7 elderly adults, age: 64±8 years) with no history of diabetes, hypertension, and neurological diseases participated in this study. All participants read and signed an informed consent approved by the Ethics in Research Committee of the Hospital das Clínicas, Faculdade de Medicina de Ribeirão Preto before participating in the study. Exclusion criteria included the presence of a pacemaker, orthosis or prosthesis incompatible with the magnetic resonance environment; claustrophobia; dementia or cognitive impairment; diabetes, hypertension and neurological diseases; and not signing the consent form.

### MRI acquisition

Experiments were performed on a 3T Philips Achieva System (Philips Achieva, The Netherlands), using an 8-channel head coil for reception and a body coil for transmission. Pulsed ASL images were acquired using a 2D single-shot EPI sequence with the following parameters: TR/TE=3000/15 ms, matrix=64×64, FOV=240×240 mm^2^, number of slices=12, slice thickness=5 mm, gap=0.5 mm, labeling plan thickness=200 mm, number of control/label pairs=40. PLDs were 1500 and 2000 ms for young and elderly adults, respectively (21). For CVR evaluation, ASL images were acquired twice: 40 control/label pairs under normocapnia and 40 control/label pairs under hypercapnia. BOLD images were acquired using a 2D single-shot EPI sequence with the following parameters: TR/TE=2000/30 ms, matrix=128×128, FOV=230x230 mm^2^, number of slices=30, slice thickness=4 mm, no gap. BOLD-CVR paradigm consisted of 5 epochs of hypercapnia (14 s each) intercalated by 6 epochs of normocapnia (30 s each). Hypercapnic epochs of 14 s were chosen to assess the temporal characteristics of the hemodynamic response, such as time-to-peak (TTP) and full-width-at-half-maximum (FWHM). ASL, under normocapnia and hypercapnia, and BOLD scans were randomized and interleaved with anatomical scans. In addition, a high-resolution 3DT1 weighted image was acquired with the following parameters: TR/TE=7/3.1 ms, matrix=240×240, excitation angle=8°, FOV=240×240 mm^2^, number of slices=160, slice thickness=1 mm. Total acquisition time was 40 min, including other sequences acquired for clinical diagnosis.

All participants were instructed to abstain from consuming coffee and alcohol for at least 12 h before the MRI section since both substances have a vasomotor effect. All MRI sections were performed in the morning to avoid variability due to diurnal CBF fluctuations.

### Hypercapnic challenge

**Figure 1:**
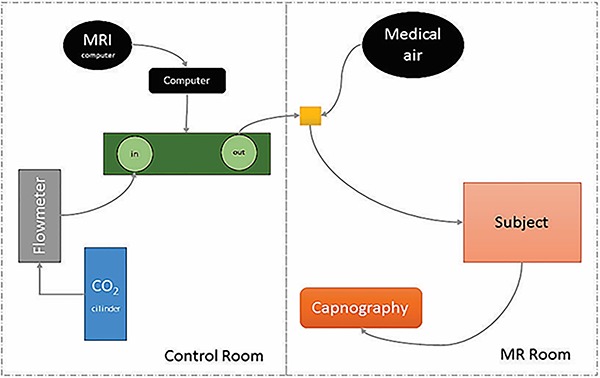
Schematic experimental setup. CO_2_ goes through the flowmeter into a device consisting of micro-valves controlled by a software and synchronized with image acquisition. When the valves open, CO_2_ goes through a rubber tube into the magnetic resonance (MR) room, where it mixes with medical air. This gas mixture is delivered to the participant through a nasal cannula, which also has a connection with an MR-compatible vital sign monitor (capnography) to continuously check and record the physiological parameters (end-tidal CO_2_, SpO_2_, heart rate and respiration rate) during the experiments.

Hypercapnia was achieved by inhalation of CO_2_. A device consisting of micro-controlled valves was developed in the Departamento de Física (Faculdade de Filosofia, Ciências e Letras de Ribeirão Preto, Universidade de São Paulo, Brazil) to deliver CO_2_ mixed with medical air through a nasal cannula. The device was controlled using Presentation software (Neurobehavioral Systems Inc., USA), and synchronized with image acquisition. Figure 1 shows the experimental setup. Briefly, CO_2_ goes into the device through the flowmeter. The opening of the valves is triggered with MR pulses. When the valves open, CO_2_ goes through a rubber tube into the MR room, where it mixes with medical air in a T-shaped connector. Then, this gas mixture is delivered to the participant through a nasal cannula, which also has a connection with an MR-compatible vital sign monitor (Veris MR, Medrad Inc., USA). The delay between opening of the valves and delivery of the gas mixture to the participant was less than 2 s; therefore, the first image acquired in each sequence under hypercapnia was excluded from analysis. Although the experiment was set up to deliver a gas mixture with 5% of CO_2_, participants were also able to breathe room air through the nasal cannula in addition to the gas mixture. We used this setup instead of a mask to minimize participant's discomfort. Moreover, the MR-compatible vital sign monitor was used to continuously check and record the physiological parameters (end-tidal CO_2_, SpO_2_, heart rate and respiration rate) during the experiments.

### Data analysis

Data processing was performed using BrainVoyager QX (Brain Innovation BV, The Netherlands), Statistical Parametric Mapping (SPM12, University College London, UK), an open-source toolbox for ASL image (ASLtbx) (27), and routines developed by our group in Matlab (The MathWorks, Inc., USA). First, ASL and BOLD images were corrected for head motion. For ASL data, a separate realignment of the control and label image series was performed (27). Data was excluded if the translational or rotational movement was greater than 1 mm or 1°, respectively. Data from one elderly participant was excluded due to incorrigible movement artifacts.

After realignment, the subtraction of both ASL phases (control - label) was performed to obtain perfusion-weighted maps for both conditions, normocapnia and hypercapnia. Spatial SNR of the global perfusion signal was calculated as the ratio between the mean perfusion signal in the brain and the background noise level, which was estimated as the standard deviation of the noise signal obtained in two regions of interest outside the brain.

CBF maps were calculated using a model described by Golay et al. (28), assuming some parameters: labeling efficiency, 0.95; brain/blood partition coefficient, 0.98 for GM, and 0.84 for white matter; apparent tissue relaxation longitudinal time, 1100 ms, and arterial blood longitudinal relaxation time, 1680 ms. With quantitative normocapnic (CBF_0_) and hypercapnic (CBF) values, ASL-CVR was calculated using Equation 1.

(1) $$CVR=100\times \frac{\frac{\Delta CBF}{CBF_{0}}}{\Delta EtCO_{2}}$$

CBF and ASL-CVR maps were spatially smoothed with a Gaussian filter (FWHM=4 mm), coregistered to the anatomical images and normalized to the MNI standard space. A voxel-wise analysis between groups for CBF and ASL-CVR maps was performed using a 2-sample *t*-test (P<0.001, uncorrected) and no cluster size threshold.

For BOLD images, temporal correction between slices, temporal filtering using a high-pass filter of 0.01 Hz, spatial smoothing with a Gaussian filter (FWHM=4 mm), coregistration to anatomical images and normalization to the MNI standard space were performed in this order. Then, a general linear model was used to obtain statistical maps in response to hypercapnia. Hemodynamic responses were fitted to a Gaussian function to obtain their amplitude and time-to-peak. BOLD-CVR was calculated as the signal amplitude divided by the increase in end-tidal CO_2_ (ΔEtCO_2_).

CBF, ASL-CVR, and BOLD amplitude, TTP, FWHM and CVR values were obtained for GM. Data are reported as means±SD. Statistical analysis was performed using ANOVA and *t*-test, or corresponding non-parametric tests. Statistical significance was set at P<0.05 (two-sided).

## Results

### Baseline CBF

**Figure 2:**
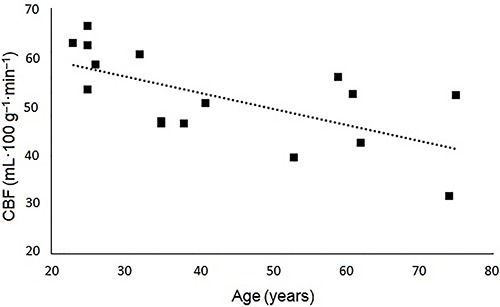
Decrease in normocapnic cerebral blood flow (CBF) with aging (linear fit, slope=-0.33, r=-0.64, P=0.02).

**Figure 3:**
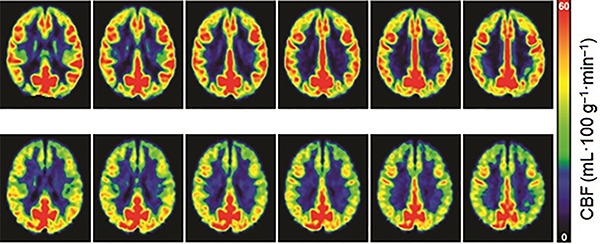
Averaged normocapnic cerebral blood flow (CBF) maps for young (upper row) and elderly healthy adults (bottom row).

During normocapnia, physiological parameters were within normal limits for all subjects. Average end-tidal CO_2_ was 35±3 mmHg. Perfusion images showed high SNR (13.1±3.6). Moreover, mean CBF across the entire GM decreased with age (linear fit, slope=-0.33, r=-0.64, P=0.02; Figure 2), but no gender difference was observed. Separating the results by age groups, CBF in GM was significantly reduced for elderly (mean: 46, median: 48, ±SD: 9 mL·100 g^-1^·min^-1^) compared to young adults (mean: 57, median: 59, ±SD: 8 mL·100 g^-1^·min^-1^; P=0.02). Figure 3 shows averaged CBF maps for both groups.

**Figure 4:**
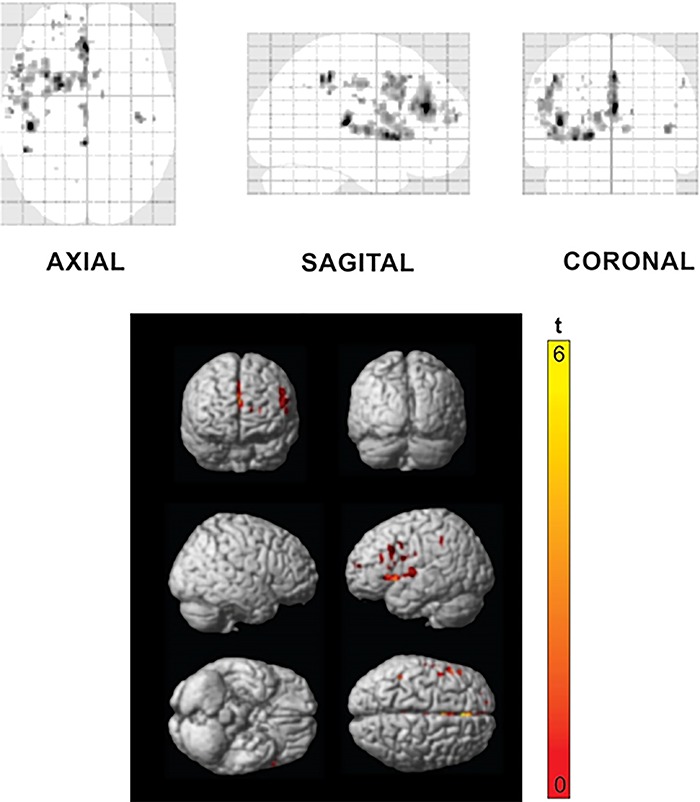
Statistical Parametric Map of differences in regional cerebral blood flow (CBF) between young and elderly adults under normocapnia. The colormap indicates brain regions where CBF is higher in young adults compared to the elderly adults (P<0.001, *t*-test uncorrected for multiple comparisons).

A voxelwise analysis using a 2-sample *t*-test showed age-related decreases in CBF for different brain regions in both hemispheres (Figure 4): inferior, middle and superior frontal gyri; precentral and postcentral gyri; superior temporal gyrus; cingulate gyri; insula, putamen, caudate, and inferior parietal lobule (supramarginal gyrus). No region showed significantly increased CBF in elderly.

### Cerebrovascular reactivity

For CVR evaluation, subjects were subjected to a hypercapnic challenge. During hypercapnia, physiological parameters stayed within normal limits for all subjects, except for end-tidal CO_2_ that significantly increased for both experiments, with pulsed ASL (ΔEtCO_2_=6±3 mmHg) and with BOLD-MRI (ΔEtCO_2_=7±3 mmHg). Moreover, CBF and BOLD signal increased in the entire GM for both groups. However, CVR values were significantly lower for elderly subjects (Table 1; P<0.05). No regional or gender differences were observed. Moreover, evaluating the temporal dynamics of CBF under hypercapnia, no habituation to CO_2_ inhalation was observed.

**Table 1:**
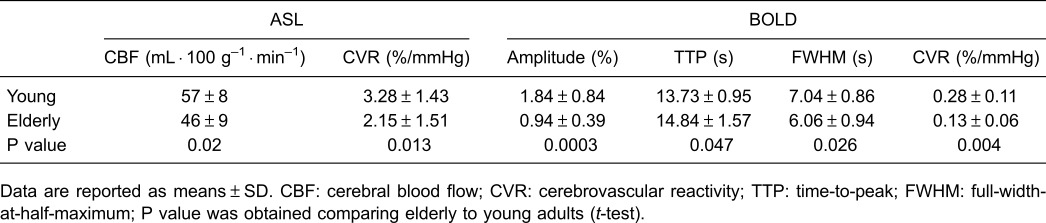
Pulsed arterial spin labeling (ASL) and blood oxygenation level-dependent (BOLD) contrast results from gray matter in hypercapnic condition.

**Figure 5:**
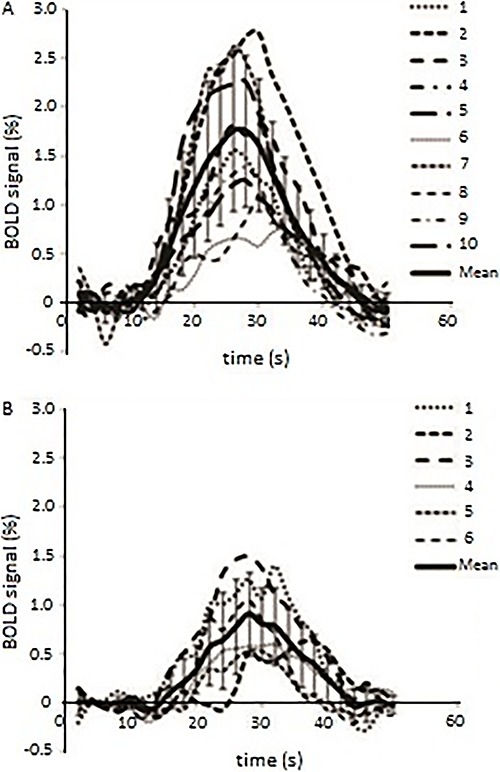
Blood oxygenation level-dependent (BOLD) contrast response to hypercapnia for (*A*) young and (*B*) elderly adults, showing a great inter-subject variability.

From BOLD images, it was possible to obtain the hemodynamic response evoked by hypercapnia. There was a great inter-subject variability regarding response amplitude for both groups (Figure 5A and B). Significantly reduced amplitude (P=0.0003) and FWHM (P=0.026), and increased time-to-peak (P=0.047) were observed for elderly participants in GM. However, no regional differences were observed for both groups, although there was a tendency of time-to-peak to be greater in posterior regions for elderly participants (P=0.06). Taken together, the results suggest that cerebrovascular reactivity is reduced and slower in healthy aged subjects.

**Figure 6:**
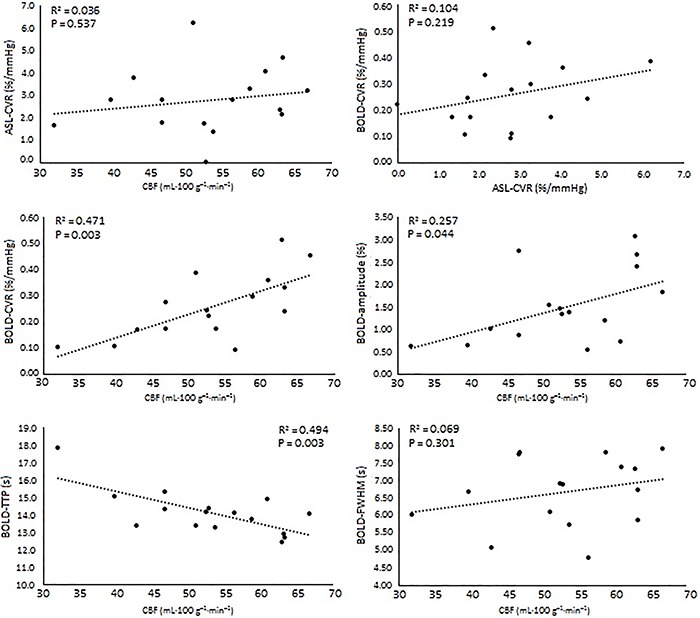
Relationship between blood oxygenation level-dependent (BOLD) contrast and pulsed arterial spin labeling (ASL) measurements across the gray matter for all young and elderly subjects (n=16). CVR: cerebrovascular reactivity; TTP: time-to-peak; FWHM: full-width-at-half-maximum.

Moreover, no significant correlation was observed between ASL-CVR and BOLD-CVR, and between ASL-CVR and baseline CBF for global GM. However, a significant positive correlation was observed between BOLD response parameters (CVR, amplitude, TTP and FWHM) and baseline CBF for GM (Figure 6).

## Discussion

The protocol used in this study allowed the quantification of CBF as well as assessment of CVR in response to a hypercapnic challenge in young and elderly adults. Decreased CBF and CVR were observed in healthy elderly compared with healthy young adults, suggesting some degree of vascular impairment with aging.

To assess baseline CBF, we used a pulsed ASL method available for clinical use in the hospital where images were acquired. CBF maps showed satisfactory SNR (29). Although pulsed ASL has the advantages of high labeling efficiency and small energy deposition, it is highly dependent on AAT, which may cause errors in CBF quantification if PLD is not chosen appropriately (15,20). For CBF quantification, ideally, the PLD has to be longer than the longest value of AAT so that all labeled blood is delivered to the tissue before image acquisition. However, in this case, SNR is compromised since ASL signal decays with T_1_ after labeling. On the other hand, if PLD is not long enough, some brain areas may present a low signal (no labeled blood), or very high signal (labeled blood still in large vessels). In both cases, CBF quantification is not accurate. Therefore, two PLD values were used, 1500 and 2000 ms, for young and elderly adults, respectively, as recommended by the ISMRM Perfusion Study Group and the European Consortium for ASL in Dementia (21).

For young adults, baseline CBF and its increase in response to hypercapnia was consistent with other studies published recently (3,30,31), showing the ability of the technique to detect global increases in CBF associated with a vasoactive stimulus. Elderly volunteers showed reduced baseline CBF in different brain regions. These results are in agreement with previous studies using ASL methods (4,22,23,25,32,33). Although some studies reported no CBF differences with advanced age (4,24), Liu et al. (22) established that CBF is diminished in elderly even when accounting for major confounding effects from vascular alterations. Moreover, the CBF decrease in the elderly has been associated with degenerative changes in microvasculature (33) and linked with subcortical white matter health (23), which may have implications for the mechanisms of neurodegeneration (34).

To evaluate CVR, we used a hypercapnic challenge in combination with pulsed ASL and BOLD-MRI. Recently, Zhou and colleagues reported similar global CVR obtained with ASL and BOLD-MRI, showing their complementary characteristics (35). In the present study, both methods showed impaired GM CVR with advanced age, and BOLD-MRI showed a slower hemodynamic response to CO_2_. These results are consistent with previous reports in the literature and have been associated with age-related arteriosclerosis and vascular stiffening (1,10,36,37).

Although the gas challenge used in our BOLD experiment is shorter than the ones reported in previous studies, 14-s epochs of hypercapnia evoked BOLD responses comparable to the results from a study that used a precise control of end-tidal carbon dioxide and oxygen, and epochs of 80 s (38). The BOLD amplitude and CVR values were very similar between our study and that from Mark et al. (38). Also, the maximum EtCO_2_ reached during a 14-s hypercapnia (BOLD experiment) was not significantly different from the one observed during a 4-min hypercapnia (ASL experiment). Moreover, with this protocol, we were able to assess the temporal characteristics of BOLD response and observe that there is a significant positive correlation between each BOLD parameter and the baseline CBF. These results confirm a smaller capacity of vasodilation in elderly adults.

Moreover, it has been shown that normal aging is associated with changes in cerebrovascular function, structure and cellular metabolism (3,39). Even in the absence of Alzheimer's disease and other degenerative illness, aging is accompanied by a significant decline in memory, language, and motor functions (1,2,11). Therefore, cerebral perfusion impairment observed, for example, in middle and inferior frontal gyri, and precentral gyrus may be associated with vulnerability to neurological disorders in the elderly.

However, the present study has some limitations. First, it is a cross-sectional study limited by great inter-subject variability. A longitudinal study would be better in this case, but it would take years to be done. Second, group sizes are small, implying that generalizations should be made with prudence. Especially for the elderly group, participant recruitment was difficult because diabetes and hypertension were exclusion criteria, and both are associated with perfusion impairment (40). Therefore, this is a pilot study showing the feasibility of using readily available ASL sequence to assess CBF and CVR, but more data is needed to better assess differences between the two groups. Third, for the hypercapnic challenge, CO_2_ delivery was not strictly controlled, since participants were allowed to breathe room air. This method was chosen considering the participant's comfort; and although it may increase intersubject variability, normalization using ETCO_2_ was done to minimize this effect. Finally, pulsed ASL has a high dependency on AAT, making pseudo-continuous ASL or multi-PLD methods better choices for perfusion assessment. However, pulsed ASL was chosen because it was the method available for clinical use in the hospital where images were acquired.

In conclusion, CBF and CVR were successfully evaluated using an ASL sequence available for clinical use and hypercapnic challenge that causes minimal or no discomfort for the participants, which may be important for studies with patients. Age-related decreases in CBF and CVR were observed in the cerebral cortex, showing altered vascular reserve with aging. Moreover, CBF impairment was observed in some cortical and subcortical regions, which may be related to vulnerability for neurological disorders.

## References

[1] Gauthier CJ, Madjar C, Desjardins-Crépeau L, Bellec P, Bherer L, Hoge RD (2013). Age dependence of hemodynamic response characteristics in human functional magnetic resonance imaging. Neurobiol Aging.

[2] Asllani I, Habeck C, Borogovac A, Brown TR, Brickman AM, Stern Y (2009). Separating function from structure in perfusion imaging of the aging brain. Hum Brain Mapp.

[3] Bangen KJ, Restom K, Liu TT, Jak AJ, Wierenga CE, Salmon DP (2009). Differential age effects on cerebral blood flow and BOLD response to encoding: associations with cognition and stroke risk. Neurobiol Aging.

[4] Preibisch C, Sorg C, Förschler A, Grimmer T, Sax I, Wohlschläger AM (2011). Age-related cerebral perfusion changes in the parietal and temporal lobes measured by pulsed arterial spin labeling. J Magn Reson Imaging.

[5] Leoni RF, Mazzetto-Betti KC, Silva AC, Dos Santos AC, de Araujo DB, Leite JP (2012). Assessing cerebrovascular reactivity in carotid steno-occlusive disease using MRI BOLD and ASL techniques. Radiol Res Pract.

[6] Kontos HA, Raper AJ, Patterson JL (1977). Analysis of vasoactivity of local pH, PCO_2_ and bicarbonate on pial vessels. Stroke.

[7] Iadecola C (1992). Does nitric oxide mediate the increases in cerebral blood flow elicited by hypercapnia?. Proc Natl Acad Sci U S A.

[8] Ito H, Kanno I, Ibaraki M, Hatazawa J, Miura S (2003). Changes in human cerebral blood flow and cerebral blood volume during hypercapnia and hypocapnia measured by positron emission tomography. J Cereb Blood Flow Metab.

[9] Duong TQ, Iadecola C, Kim SG (2001). Effect of hyperoxia, hypercapnia, and hypoxia on cerebral interstitial oxygen tension and cerebral blood flow. Magn Reson Med.

[10] Cantin S, Villien M, Moreaud O, Tropres I, Keignart S, Chipon E (2011). Impaired cerebral vasoreactivity to CO_2_ in Alzheimer's disease using BOLD fMRI. Neuroimage.

[11] Richiardi J, Monsch AU, Haas T, Barkhof F, Van de Ville D, Radü EW (2014). Altered cerebrovascular reactivity velocity in mild cognitive impairment and Alzheimer's disease. Neurobiol Aging.

[12] Raut RV, Nair VA, Sattin JA, Prabhakaran V (2016). Hypercapnic evaluation of vascular reactivity in healthy aging and acute stroke via functional MRI. Neuroimage Clin.

[13] Haight TJ, Bryan RN, Erus G, Davatzikos C, Jacobs DR, D'Esposito M (2015). Vascular risk factors, cerebrovascular reactivity, and the default-mode brain network. Neuroimage.

[14] Wintermark M, Sesay M, Barbier E, Borbély K, Dillon WP, Eastwood JD (2005). Comparative overview of brain perfusion imaging techniques. J Neuroradiol.

[15] Barbier EL, Lamalle L, Décorps M (2001). Methodology of brain perfusion imaging. J Magn Reson Imaging.

[B16] Golay X, Petersen ET, Zimine I, Lim TC (2007). Arterial spin labeling: a one-stop-shop for measurement of brain perfusion in the clinical settings. Conf Proc IEEE Eng Med Biol Soc.

[17] Paiva FF, Tannús A, Silva AC (2007). Measurement of cerebral perfusion territories using arterial spin labeling. NMR Biomed.

[18] Pollock JM, Tan H, Kraft RA, Whitlow CT, Burdette JH, Maldjian JA (2009). Arterial spin-labeled MR perfusion imaging: clinical applications. Magn Reson Imaging Clin N Am.

[19] Uchihashi Y, Hosoda K, Zimine I, Fujita A, Fujii M, Sugimura K (2011). Clinical application of arterial spin-labeling MR imaging in patients with carotid stenosis: quantitative comparative study with single-photon emission CT. AJNR Am J Neuroradiol.

[20] MacIntosh BJ, Lindsay AC, Kylintireas I, Kuker W, Günther M, Robson MD (2010). Multiple inflow pulsed arterial spin-labeling reveals delays in the arterial arrival time in minor stroke and transient ischemic attack. AJNR Am J Neuroradiol.

[21] Alsop DC, Detre JA, Golay X, Günther M, Hendrikse J, Hernandez-Garcia L (2015). Recommended implementation of arterial spin-labeled perfusion MRI for clinical applications: A consensus of the ISMRM Perfusion Study Group and the European Consortium for ASL in Dementia. Magn Reson Med.

[22] Liu Y, Zhu X, Feinberg D, Guenther M, Gregori J, Weiner MW (2012). Arterial spin labeling MRI study of age and gender effects on brain perfusion hemodynamics. Magn Reson Med.

[23] Chen JJ, Rosas HD, Salat DH (2013). The relationship between cortical blood flow and sub-cortical white-matter health across the adult age span. PLoS One.

[24] Rusinek H, Brys M, Glodzik L, Switalski R, Tsui WH, Haas F (2011). Hippocampal blood flow in normal aging measured with arterial spin labeling at 3T. Magn Reson Med.

[25] Chen JJ, Rosas HD, Salat DH (2011). Age-associated reductions in cerebral blood flow are independent from regional atrophy. Neuroimage.

[26] Hutchison JL, Lu H, Rypma B (2013). Neural mechanisms of age-related slowing: the ΔCBF/ΔCMRO2 ratio mediates age-differences in BOLD signal and human performance. Cereb Cortex.

[27] Wang Z, Aguirre GK, Rao H, Wang J, Fernández-Seara MA, Childress AR (2008). Empirical optimization of ASL data analysis using an ASL data processing toolbox: ASLtbx. Magn Reson Imaging.

[28] Golay X, Petersen ET, Hui F (2005). Pulsed star labeling of arterial regions (PULSAR): a robust regional perfusion technique for high field imaging. Magn Reson Med.

[29] Vidorreta M, Wang Z, Rodríguez I, Pastor MA, Detre JA, Fernández-Seara MA (2013). Comparison of 2D and 3D single-shot ASL perfusion fMRI sequences. Neuroimage.

[30] Lee C, Lopez OL, Becker JT, Raji C, Dai W, Kuller LH (2009). Imaging cerebral blood flow in the cognitively normal aging brain with arterial spin labeling: implications for imaging of neurodegenerative disease. J Neuroimaging.

[31] Tancredi FB, Gauthier CJ, Madjar C, Bolar DS, Fisher JA, Wang DJ (2012). Comparison of pulsed and pseudocontinuous arterial spin-labeling for measuring CO_2_ -induced cerebrovascular reactivity. J Magn Reson Imaging.

[32] Parkes LM, Rashid W, Chard DT, Tofts PS (2004). Normal cerebral perfusion measurements using arterial spin labeling: reproducibility, stability, and age and gender effects. Magn Reson Med.

[33] Wagner M, Jurcoane A, Volz S, Magerkurth J, Zanella FE, Neumann-Haefelin T (2012). Age-related changes of cerebral autoregulation: new insights with quantitative T2'-mapping and pulsed arterial spin-labeling MR imaging. AJNR Am J Neuroradiol.

[34] Popa-Wagner A, Buga AM, Popescu B, Muresanu D (2015). Vascular cognitive impairment, dementia, aging and energy demand. A vicious cycle. J Neural Transm.

[35] Zhou Y, Rodgers ZB, Kuo AH (2015). Cerebrovascular reactivity measured with arterial spin labeling and blood oxygen level dependent techniques. Magn Reson Imaging.

[36] Flück D, Beaudin AE, Steinback CD, Kumarpillai G, Shobha N, McCreary CR (2014). Effects of aging on the association between cerebrovascular responses to visual stimulation, hypercapnia and arterial stiffness. Front Physiol.

[37] Bhogal AA, De Vis JB, Siero JC, Petersen ET, Luijten PR, Hendrikse J (2016). The BOLD cerebrovascular reactivity response to progressive hypercapnia in young and elderly. Neuroimage.

[38] Mark CI, Slessarev M, Ito S, Han J, Fisher JA, Pike GB (2010). Precise control of end-tidal carbon dioxide and oxygen improves BOLD and ASL cerebrovascular reactivity measures. Magn Reson Med.

[39] Restom K, Bangen KJ, Bondi MW, Perthen JE, Liu TT (2007). Cerebral blood flow and BOLD responses to a memory encoding task: a comparison between healthy young and elderly adults. Neuroimage.

[40] Leoni R, Paiva FF, de Araujo DB, Silva AC (2009). Reduced cerebrovascular reactivity to CO_2_ induced by hypertension and age: an arterial spin labeling study in rats. J Cerebral Blood Flow Metab.

